# Fibrogenic Pathways in Metabolic Dysfunction Associated Fatty Liver Disease (MAFLD)

**DOI:** 10.3390/ijms23136996

**Published:** 2022-06-23

**Authors:** Pallavi Subramanian, Jochen Hampe, Frank Tacke, Triantafyllos Chavakis

**Affiliations:** 1 Institute for Clinical Chemistry and Laboratory Medicine, University Hospital and Faculty of Medicine, Technische Universität Dresden, 01307 Dresden, Germany; triantafyllos.chavakis@uniklinikum-dresden.de; 2 Department of Internal Medicine I, University Hospital and Faculty of Medicine, Technische Universität Dresden, 01307 Dresden, Germany; jochen.hampe@uniklinikum-dresden.de; 3 Department of Hepatology & Gastroenterology, Campus Virchow-Klinikum and Campus Charité Mitte, Charité Universitätsmedizin Berlin, 13353 Berlin, Germany; frank.tacke@charite.de; 4 Paul Langerhans Institute Dresden, Helmholtz Zentrum München, University Hospital and Faculty of Medicine, Technische Universität Dresden, 01307 Dresden, Germany; 5 German Center for Diabetes Research, 85764 Neuherberg, Germany

**Keywords:** nonalcoholic fatty liver disease, metabolic associated fatty liver disease, nonalcoholic steatohepatitis, hepatic stellate cells, fibrosis, hepatocyte, macrophage

## Abstract

The prevalence of nonalcoholic fatty liver disease (NAFLD), recently also re-defined as metabolic dysfunction associated fatty liver disease (MAFLD), is rapidly increasing, affecting ~25% of the world population. MALFD/NAFLD represents a spectrum of liver pathologies including the more benign hepatic steatosis and the more advanced non-alcoholic steatohepatitis (NASH). NASH is associated with enhanced risk for liver fibrosis and progression to cirrhosis and hepatocellular carcinoma. Hepatic stellate cells (HSC) activation underlies NASH-related fibrosis. Here, we discuss the profibrogenic pathways, which lead to HSC activation and fibrogenesis, with a particular focus on the intercellular hepatocyte–HSC and macrophage–HSC crosstalk.

## 1. Introduction

The prevalence of metabolic dysfunction-associated fatty liver disease (MAFLD), previously designated nonalcoholic fatty liver disease (NAFLD), is rapidly growing and affects approximately 25% of the world population [1,2,3]. The highest prevalence of MAFLD is in South America and the Middle East and the lowest is in Africa [3]. In addition to the clinical burden, the economic burden caused by MAFLD is substantial [4]. MAFLD comprises several liver pathologies ranging from simple hepatic steatosis to the more severe disease form, known as nonalcoholic steatohepatitis (NASH) [5]. NASH is characterized by hepatocyte death and inflammation (together also summarized as necroinflammation) and may further progress to fibrosis, and further adverse hepatic outcomes, such as cirrhosis, hepatocellular carcinoma and liver failure [6]. Furthermore, more than 80% of people with NASH are obese, >40% have type 2 diabetes mellitus and ~70% have dyslipidemia, thereby supporting the notion that NASH is the liver manifestation of metabolic syndrome [3,7]. The importance of NASH as a health burden is further illustrated by the fact that NASH is the fastest growing and the second most common indication for liver transplantation in the United States [8]. Currently, no approved pharmacotherapies exist for NASH, partly due to an incomplete understanding of the underlying disease mechanisms [6,9]. Fibrosis severity is an important indicator of both liver-related and all-cause mortality during NASH [7,10,11]. The risk of liver-related death is significantly higher in NASH patients who have F3 or F4 stage fibrosis, as compared to those with little to no fibrosis [7].

Liver resident hepatic stellate cells (HSC), a non-parenchymal cell population of mesenchymal origin, give rise to the majority of myofibroblasts in various rodent models of liver fibrosis, including a diet-induced MAFLD/NASH model [12,13]. Once activated, the quiescent HSCs transdifferentiate into extracellular matrix (ECM)-producing, highly proliferative myofibroblasts (MFB) [14]. The excess accumulation of ECM combined with its inadequate degradation leads to hepatic fibrosis development [6,15]. HSC activation is a multicellular response regulated by liver-resident and non-resident cells such as hepatocytes, Kupffer cells (liver resident macrophages) and recruited immune cells, respectively [15,16,17,18]. These dynamic cellular interactions may include either direct cell–cell crosstalk, such as hepatocyte–HSC communication, or indirect interactions, for instance, hepatocyte–immune cell crosstalk that ultimately leads to HSC activation. In this review, we will summarize pathways, by which hepatocyte–HSC and macrophage–HSC crosstalk may regulate HSC activation and hence fibrosis development. We will also discuss the pathological and physiological functions of HSCs in the liver.

## 2. Functions and Biology of Hepatic Stellate Cells

In 1876, Carl von Kupffer first described HSCs in a letter to his colleague Waldeyer, in which he named these cells ‘Sternzellen or stellate cells’ [19,20,21]. HSCs, previously known as perisinusoidal cells, lipocytes, Ito cells and fat storing cells, are liver resident non-parenchymal cells that perform a wide range of physiological and pathological functions [14,20,21,22,23]. In the normal liver, HSCs represent 5–8% of the total hepatic cells and are localized in the space of Disse between sinusoidal endothelial cells and the basolateral surface of hepatocytes [14,20,21,22,23,24]. In human liver, there are approximately 10 HSCs per 100 hepatocytes and in rat liver approximately 13 HSCs per 100 parenchymal cells [20]. HSCs have multiple cytoplasmic extensions or processes which facilitate intercellular transport of soluble factors and assist in making contact with the surrounding cells, such as hepatocytes, endothelial cells and nerve endings [22,24,25].

A distinct characteristic feature of quiescent HSCs in healthy liver is the cytoplasmic storage of retinoid (vitamin A and its metabolites) within lipid droplets [14,22,23,24,26,27]. Approximately 80–90% of hepatic retinoids are stored in HSC lipid droplets [22,23]. HSCs play a major role in the uptake, storage and metabolism of vitamin A or retinol [20,23,28]. Dietary retinol once absorbed in the gut is esterified to retinyl esters, packaged in chylomicrons and transported to the liver, where hepatocytes take up the retinol-containing chylomicrons [22,23,29]. Upon uptake by hepatocytes, the retinyl esters are metabolized to retinol and subsequently transferred to HSCs, with the help of the cellular retinol binding protein (CRBP-1) [23,30,31]. Within the HSCs, the retinol may have different fates: It can be esterified by the action of lecithin retinol acyltransferase (LRAT) for storage in lipid droplets, attached to retinol binding protein (RBP) for secretion or oxidized by alcohol dehydrogenase (ADH) to produce retinal [21,23,29,30,32]. Retinal can be further converted to the transcriptional regulator retinoic acid by the function of retinal dehydrogenase (RALDH) [29,33]. The loss of lipid droplets is considered a hallmark of HSC activation; however, there is no consensus on the function of retinoids in the context of HSC activation and fibrogenesis, as several studies have reported rather contradictory findings [34,35,36,37,38,39,40,41,42,43,44,45,46].

Quiescent HSCs support the steady state maintenance of the liver architecture by producing ECM concurrently with the production of ECM-degrading matrix metalloproteinases [29], while disruption of this balance, for instance, by metabolic insults, leads to HSC activation and fibrosis [15]. Under physiological conditions, approximately 0.5% of the liver wet weight is composed of ECM proteins [20]. The space of Disse, contains small amounts of ECM proteins; these ECM proteins support physiological homeostatic functions of HSCs, hepatocytes and endothelial cells, partly by signaling via integrins [14,20,24,47,48,49,50,51]. The composition of the ECM present in the space of Disse is broadly subdivided into collagens such as type I, III, IV and V, proteoglycans such as heparin sulfate, decorin and biglycan, structural glycoproteins such as laminin and fibronectin, and the free glycosaminoglycan hyaluronan [20]. During fibrosis, in addition to enhanced production of ECM by transdifferentiated MFBs, the composition of the ECM is also altered with upregulation of wound healing associated ECM and high level of crosslinks making the ECM resistant to degradation, thereby contributing to the difficulties of reversing advanced fibrosis [15]. Interestingly, single cell RNA sequencing studies performed in human and mouse livers have identified heterogeneity within the MFB population of fibrotic and cirrhotic livers [52,53,54,55]. Furthermore, alterations in chemokine expression are evident between resting HSCs and activated MFBs [52]. Compared to resting HSCs, MFBs induced in the CCL_4_ model, express high levels of chemokine (C-X-C motif) ligand 1 (CXCL1), whereas the expression of the chemokines chemokine (C-C motif) ligand 2 (CCL2) and chemokine (C-X-C motif) ligand 12 (CXCL12) is similar in both cell types [52].

In NASH, HSCs contribute to approximately 80–95% of the ECM-producing MFBs, as shown in multiple mouse models of fibrosis [13]. HSC activation is triggered by cellular networks consisting of liver-resident and non-resident cells such as hepatocytes, Kupffer cells (liver resident macrophages) and recruited immune cells such as monocytes and monocyte-derived macrophages or lymphocytes [16]. Multiple factors such as immune cell-derived inflammatory and fibrogenic cytokines (e.g., PDGF, VEGF, TGFβ), hedgehog ligands, apoptotic bodies, elevated levels of reactive oxygen species (ROS) and bioactive lipid mediators are known to promote HSC activation either directly or indirectly via various cell surface, nuclear and cytoplasmic receptors and pathways [15,16,21,24,56]. Transforming growth factor-β (TGFβ) is a potent profibrogenic cytokine; TGFβ induced activation of HSCs leads to the downstream activation of predominantly SMAD2/SMAD3 proteins, which in turn promote the transcriptional upregulation of collagen type I and III [21,24,28]. Furthermore, TGFβ-induced HSC activation involves extracellular signal regulated kinase (ERK), p38 and c-jun N-terminal kinase (JNK) [21,28]. TGFβ signaling enhances several NADPH oxidase enzymes, particularly, NOX1, NOX2, NOX4 and NOX5, to increase H_2_O_2_ synthesis and collagen production [28]. Other inducers of NOX enzymes in HSCs include leptin, PDGF and angiotensin II [28]. Moreover, the Hedgehog (Hh) signaling pathway has been implicated in HSC activation and NASH [21,24,28,57,58]. Hh ligands activate their cell surface receptor Patched (PTCH), thereby relieving the inhibitory action of PTCH on Smoothened (SMO), leading to its activation [59]. Once active, SMO promotes the nuclear translocation of the transcription factors GLI1, GLI2 and GLI3 ultimately resulting in Hh target gene expression [59]. PTCH heterozygous mice fed a methionine and choline deficient diet exhibited enhanced Hh activation, elevated levels of osteopontin and exaggerated fibrosis development, as compared to wild type mice [60]. Moreover, HSCs express various nuclear transcription factors, such as farnesoid X receptor (FXR), liver X receptor (LXR), peroxisome proliferator activated receptor (PPAR)δ and PPARγ; these receptors are known to negatively regulate HSC activation and fibrosis development [21,24]. Interestingly, activation of the lipogenic transcription factor sterol regulatory element binding protein (SREBP)-1C reverses HSC activation [28]. Recently, the transcription factors GATA-binding factor 6 (GATA6) and transcription factor 21 (Tcf21) were identified as factors inhibiting profibrotic HSCs, thereby promoting liver fibrosis regression [61,62]. Once activated, HSCs reprogram their cellular metabolism to meet the energy demand of the highly proliferative, ECM-producing MFB phenotype [28,63]. During HSC activation, aerobic glycolysis is upregulated, while gluconeogenesis and lipogenesis pathways are suppressed; this metabolic perturbation is regulated by the Hh pathway and hypoxia inducible factor 1α (HIF1α) [57]. As a result of increased glycolysis, HSCs accumulate lactate, which is required to invoke a global rewiring of HSC gene expression and further promote cell proliferation and fibrogenic activities [57]. Despite the robust increase in glycolysis during HSC activation, there is also an upregulation of oxidative phosphorylation, which is also evident from the increase in the number and activity of mitochondria during HSC activation [57,64]. Furthermore, glutaminolysis has been shown to regulate the activation and transdifferentiation of HSCs [58,65]. Glutaminolysis is a two-step reaction, in which glutamine is converted to α-ketoglutarate, the latter of which is an important intermediate of the tricarboxylic acid (TCA) cycle [63]. Therefore, α-ketoglutarate, the product of glutaminolysis, promotes the replenishment of the TCA cycle and enhances its activity, thereby fulfilling the high bioenergetic demand during HSC activation [58]. Furthermore, autophagy is increased in activated HSCs and generates fatty acids by cleavage of retinyl esters from the lipid droplets [21,28,66,67]. HSC-specific deficiency of the autophagy regulator, autophagy-related protein 7 (ATG7), results in reduced fibrosis development and ECM accumulation following liver injury in mice [66]. Moreover, the reduced HSC activation seen in autophagy-deficient HSCs could be partially overcome by the addition of exogenous fatty acids, thereby suggesting that autophagy generates free fatty acids to fuel cellular activation [28,66]. Interestingly, in mice, HSCs that lack retinoid containing lipid droplets due to LRAT deficiency can still fully undergo activation in response to injury and do not undergo spontaneous activation [34]. The paradoxical observation that loss of retinoid containing lipid droplets accompanies HSC activation in different models of liver injury [23,28], whereas the absence of lipid droplets due to LRAT deficiency does not alter HSC activation [34], cannot be readily explained. Nevertheless, it is possible that LRAT-deficient HSCs use different energy sources for activation [28,34]. These studies, however, suggest that the presence of the retinoid containing lipid droplets is not a prerequisite to maintain the quiescent HSC phenotype [34,68].

## 3. Hepatocyte–Hepatic Stellate Cell Crosstalk

Hepatocytes contribute to HSC activation both directly and indirectly. The latter indirect activation loop can be triggered by hepatocyte death, which in turn leads to inflammation and monocyte/macrophage recruitment, ultimately resulting in the secretion of proinflammatory and profibrotic cytokines by macrophages. Conversely, the direct hepatocyte–HSC crosstalk leading to HSC activation may be mediated by several stimuli, for example, hedgehog ligands [57,69], apoptotic bodies [70] or reactive oxygen species (ROS) [21,71,72]. Hepatocyte stress induced by lipid accumulation, specifically palmitic acid, promotes the release of extracellular vesicles containing profibrogenic mediators, for instance, microRNAs (miRNA) that can induce HSC activation [73,74,75]. In particular, lipid-loaded hepatocytes (mouse and human cells) release extracellular vesicles containing miR-128-3p that induces HSC activation by suppressing peroxisome proliferator-activated receptor-γ (PPAR-γ) [75]. Moreover, palmitic acid promotes inflammasome activation in mouse hepatocytes thereby sensitizing cells to lipopolysaccharide (LPS)-induced interleukin 1 beta (IL-1β) secretion [76]. IL-1β in turn can upregulate the fibrogenic molecule tissue inhibitor of metalloproteinase 1 (TIMP-1) and prolong survival of mouse HSCs [16,77]. Furthermore, uptake of apoptotic bodies deriving from hepatocyte death also causes human HSC activation [70,78]. Alternatively, apoptotic bodies from hepatocyte death can be taken up by macrophages in the process of efferocytosis triggering the release of TGFβ, a major driver of HSC activation and fibrogenesis [21]. Moreover, damage-associated molecular patterns (DAMP), which are mostly released during necrosis or necroptosis, for instance, DNA or high mobility group box-1 (HMGB1), promote pathways of HSC activation, as shown in mouse and human cells [79,80,81].

The TCA cycle intermediate succinate, secreted from hepatocytes, promotes HSC activation by activating G protein-coupled receptor 91 (GPR91) [82,83]. Sirtuin 3 (SIRT3), a key regulator of succinate dehydrogenase (SDH), is suppressed by palmitate treatment of hepatocytes; this results in decreased SDH activity and increased succinate, which can be exported out of the cell and activate HSCs via GPR91 [82]. Palmitate treatment also suppresses the SIRT3-SDH axis in HSCs (besides hepatocytes), thereby leading to increased secretion of succinate and exacerbates autocrine GPR91 activation [82].

Hepatocyte ballooning is a key histological hallmark of NASH and is closely associated with NASH and fibrosis [84,85]. Ballooned hepatocytes produce sonic hedgehog (SHH), which is known to activate HSCs [86,87]. Hedgehog signaling induces metabolic reprograming of HSCs with preferential induction of glycolysis thereby promoting the MFB phenotype [57]. Furthermore, the direct link between hepatic cholesterol and NASH is well established [88,89]. Increased cholesterol accumulation in hepatocytes promotes NASH fibrosis and HSC activation by stabilizing the transcriptional regulator TAZ (WWTR1), which further enhances the transcription and secretion of the profibrotic molecule Indian hedgehog (IHH) [69,90]. Both TAZ and IHH are increased in human livers with NASH but not with simple steatosis [69,91], suggesting that hepatocyte TAZ could play a role in promoting NAFLD disease progression, i.e., the transition from steatosis to NASH.

NOTCH is a developmental pathway, which functions in promoting hepatocyte progenitor differentiation towards cholangiocytes [92]. NOTCH signaling occurs via juxtacrine interactions between cells expressing notch receptors, NOTCH1-NOTCH4, and cells expressing cell surface NOTCH ligands such as Jagged1, Jagged2 and Delta-like ligands 1, 3 and 4 [93]. In mouse models of obesity, NOTCH signaling was found to regulate hepatic glucose production in a Forkhead box protein O1 (FOXO1)-dependent manner [93]. Furthermore, in high-fat diet-fed mice, NOTCH activation upregulates de novo lipogenesis and hepatic steatosis via activating the mechanistic target of rapamycin complex 1 (mTORC1) in hepatocytes [93,94]. Hepatocyte Notch activity is almost absent in normal human liver and mildly increased in simple steatosis; however, it is significantly elevated in NASH-associated fibrosis [95]. During NASH, the Toll-like receptor 4 (TLR4)-nuclear factor kappa B (NF-κB) signaling pathway is activated in pericentral hepatocytes, which are exposed to increased levels of gut-derived LPS; this results in increased Jagged1 (JAG1) expression on the cell surface [96]. Increased JAG1 expression in turn triggers the activation of Notch receptors on neighboring hepatocytes, leading to Notch-dependent gene expression, including the synthesis and secretion of the fibrogenic factor osteopontin (OPN; gene name *Spp1*) [96]. Of note, forced activation of hepatocyte-Notch leads to higher OPN expression and secretion from hepatocytes, in a manner dependent on sex determining region Y-box 9- (Sox9, a direct Notch transcriptional target [97]); secreted OPN can drive HSC activation [95]. Therefore, the inter-hepatocyte JAG1-Notch circuit can promote HSC activation via OPN. A recent study in mice has shown that the RNA binding protein human antigen R (HuR) also regulates the JAG1-Notch axis in hepatocytes [98]. By direct mRNA interaction, HuR negatively regulates *Jag1* mRNA in the liver. Accordingly, hepatocyte-specific deletion of HuR leads to elevated levels of *Jag1*, *Sox9* and *Hes1* (Hes family basic helix–loop–helix transcription factor 1, a Notch downstream target) and *Spp1* as well as various collagen subtypes, already at steady state [98]. Furthermore, the exaggerated fibrosis development seen in hepatocyte-specific HuR deficient mice after feeding with a NASH-inducing diet was partially reduced by treatment with an OPN neutralizing antibody, therefore suggesting that HuR negatively regulates the JAG1-Notch-OPN axis in hepatocytes, thereby protecting against fibrosis [98].

Bioactive lipid mediators, such as lysophosphatidic acid (LPA) and lysophosphatidylinositol (LPI), have been shown to promote HSC activation and contribute to NASH fibrosis development [56,99]. Circulating LPI levels are elevated in patients with advanced fibrosis, as compared to healthy individuals and the LPI-G protein-coupled receptor 55 (GPR55) pathway has been implicated in HSC activation [99,100]. In HSCs, the LPI-GPR55 axis activates acetyl-coenzyme A carboxylase (ACC) to induce HSC activation and profibrotic gene expression, whereas, in hepatocytes the LPI-GPR55-ACC pathway promotes de novo lipogenesis and inhibits β-oxidation [99]. However, the exact cellular source of LPI has not been investigated in these studies. Interestingly, the presence of the *MBOAT7 rs641738C>T* risk variant, which leads to reduced membrane-bound O-acyltransferase domain containing 7 (MBOAT7) mRNA and protein levels, is associated with elevated levels of LPI in the liver and consistently with enhanced fibrosis development during NAFLD [101,102]. Furthermore, hepatic levels of MBOAT7 are suppressed during obesity, even in the absence of the *MBOAT7 rs641738C>T* risk variant [100]. Therefore, the enhanced release of LPI due to reduced MBOAT7 expression may promote HSC activation, although further investigation is needed to substantiate the mechanisms underlying this crosstalk.

## 4. Macrophage–Hepatic Stellate Cell Crosstalk

Various immune cells, including macrophages, natural killer cells, T and B lymphocytes and neutrophils, infiltrate into the NAFLD liver and may orchestrate both development of liver fibrosis and fibrosis resolution [16,17,103,104]. Here, we focus on the mechanisms of macrophage–HSC communication, which may result in activation of HSCs and fibrosis development.

Kupffer cells (KC) are yolk sac-derived, liver-resident macrophages that are self-renewing and represent the main macrophage population in the heathy liver [18]. At steady state, the KC self-renewal capacity is regulated by the repressive transcription factors MafB and cMaf [18]. During NASH, there is a substantial influx of bone marrow-derived monocytes/macrophages into the liver [105]. Activation of KCs may promote the recruitment of bone marrow derived monocytes/macrophages during early stages of NASH development [106,107]. In addition, injured hepatocytes as well as activated HSC can also foster monocyte recruitment via releasing the chemokine CCL2 [108]. Toxic lipids, such as palmitate, can activate the TLR4-MD2 complex in CD11b+ F4/80^low^ infiltrating macrophages but not in CD11b+ F4/80^high^ KCs to secrete ROS and thereby promote HSC activation [72,109]. Liver macrophages are important regulators of hepatic steatosis, inflammation and fibrosis development, as suggested by several mouse studies that engaged approaches to deplete macrophages or to block monocyte recruitment [106,107,110,111,112,113]. Recent single cell RNA sequencing studies performed in human and mouse livers have identified the presence of a specialized macrophage subpopulation namely, CD9+ TREM2+ macrophages that have been designated scar-associated macrophages, NASH-associated macrophages or lipid-associated macrophages [55,114,115]. The CD9+ TREM2+ scar-associated macrophages expand in human cirrhotic livers, accumulate in the fibrotic niche and express several fibrosis promoting genes, including *PDGFB*, promoting HSC proliferation [116], *SPP1*, inducing HSC activation [60] and *IL1B*, promoting both HSC survival and activation [16,55,77].

As mentioned above, a dominant driver of HSC activation and NASH fibrogenesis is hepatocyte death and resulting macrophage recruitment and activation [80]. Upon hepatocyte death, recruited macrophages engage in clearing apoptotic cells via efferocytosis; the latter process can suppress inflammation and induce inflammation resolution [17,117,118]. During efferocytosis, immune regulatory factors, such as TGFβ, are produced by macrophages [117]. TGFβ, however, promotes HSC activation; moreover, c-mer tyrosine kinase (MerTK) signaling in macrophages leads to TGFβ upregulation and thereby HSC activation [119,120]. Specifically, MerTK signaling in macrophages activates the ERK1/2 pathway leading to increased TGFβ expression, and myeloid cell-specific targeting of MerTK in mice fed a NASH-inducing diet partially protects from liver fibrosis development [120]. Furthermore, TLR4 signaling in HSCs sensitizes them to subsequent TGFβ signals and facilitates their activation [121]. LPS-induced TLR4 activation in quiescent HSCs downregulates the expression of the TGFβ pseudoreceptor Bambi, thereby resulting in the uncontrolled activation of HSCs by macrophage-derived TGFβ [121]. Interestingly, the serum concentration of TGFβ is elevated to similar levels in patients with simple steatosis as well as in NASH patients [122]. Furthermore, chaperone proteins, which have been implicated in NAFLD, are known to regulate TGFβ signaling [123,124]. Additionally, TGFβ signaling in HSCs can be regulated by vitamin D and the vitamin D receptor [125,126]. TGFβ induced activation of primary human HSCs is abrogated by co-treatment with vitamin D [125].

During NASH, several factors, such as cholesterol, fatty acids, oxidized low density lipoprotein (oxLDL) and other toxic lipids, promote macrophage activation and induce their proinflammatory phenotype [89,127,128]. An essential lipotoxic molecule in the context of NASH is free cholesterol (FC) [89]. Using different animal models, several studies have demonstrated the link between excess dietary cholesterol and the development of NASH [129,130,131,132,133,134]. In human livers, FC levels are increased during NASH but not in simple steatosis [89]. Moreover, cholesterol crystals are present in lipid droplets of steatotic hepatocytes during NASH; additionally, dying/dead hepatocytes, which contain cholesterol crystals, are surrounded by KCs/macrophages, thereby forming the characteristic ‘crown-like structures’ (CLS) [89]. In this setting, KCs/macrophages in the CLS may be activated by cholesterol crystals, which results in NLRP3 inflammasome activation and production of IL1β, as well as TGFβ and CCL2, which, besides exacerbating inflammation, can promote HSC activation [89]. Additionally, FC accumulation in HSCs leads to enhanced cell activation in a TLR4 dependent manner [135].

Chemokine (C-C Motif) Ligand 3 (CCL3), also known as macrophage inflammatory protein 1-alpha (MIP1α), is a ligand for the chemokine receptors, C-C chemokine receptor type 1 (CCR1) and C-C chemokine receptor type 5 (CCR5) [113]. Macrophages present in fibrotic livers secrete CCL3/MIP1α [136], which is known to directly act on HSCs to promote cell activation, proliferation and migration [137]. Moreover, chemokine (C-C motif) ligand 5 (CCL5 or RANTES), which is produced by macrophages, promotes HSC activation, proliferation and migration, as shown by treatment of HSCs with a CCL5 receptor antagonist [138]. Proinflammatory cytokines, IL-1β and tumor necrosis factor (TNF), produced by macrophages, promote the survival of activated HSCs in a NF-κB signaling pathway-dependent fashion [139]. Furthermore, macrophage derived galectin-3 induces HSC activation and fibrosis [21,24,140]. Galectin-3 is required for TGFβ-dependent cell activation; deletion of galectin-3 reduces HSC activation, collagen synthesis and liver fibrosis in a model of CCl_4_-induced liver injury [141]. Based on the central role of the HSC-macrophage crosstalk for HSC activation, it is intriguing to consider therapeutically targeting these interactions.

## 5. Conclusions and Future Perspectives

**Figure 1:**
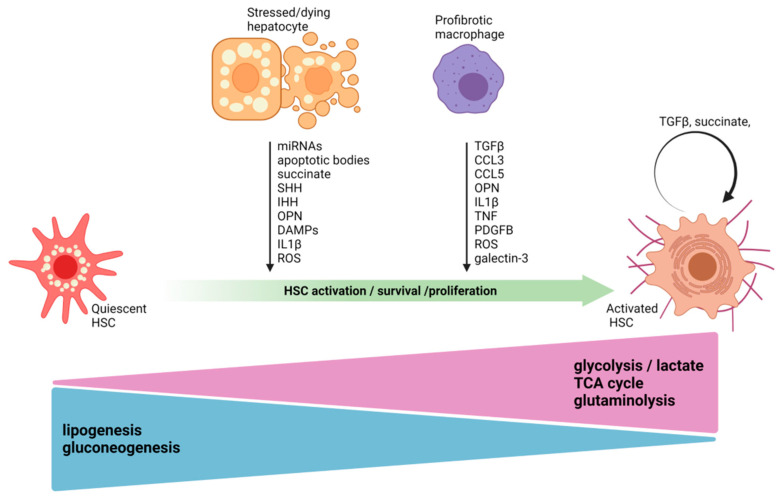
Cellular crosstalk in the context of HSC activation during NASH, based on mouse models. Quiescent HSCs in normal liver contain retinoid-rich lipid droplets and exhibit high levels of lipogenesis and gluconeogenesis. During NASH, stressed hepatocytes and macrophages can activate HSCs, which then transdifferentiate into ECM-producing, highly proliferative MFBs and contribute to hepatic fibrosis development. The lipogenesis and gluconeogenesis metabolic pathways are downregulated in activated MFBs, whereas, glycolysis, the TCA cycle and glutaminolysis are upregulated. Higher glycolysis leads to enhanced intracellular lactate accumulation, which further propagates the profibrogenic reprogramming of HSC gene expression. Stressed or dying hepatocytes and liver macrophages promote HSC activation, survival and proliferation by secreting a wide range of molecules, which include cytokines, chemokines, miRNAs, ROS, DAMPs, metabolites and apoptotic bodies. miRNA, microRNA; SHH, sonic hedgehog; IHH, Indian hedgehog; OPN, osteopontin; DAMP, damage-associated molecular patterns; ROS, reactive oxygen species; TGFβ, transforming growth factor-β; CCL3, chemokine (C-C Motif) Ligand 3; CCL5, chemokine (C-C motif) ligand 5; TNF, tumor necrosis factor; PDGFB, platelet-derived growth factor; IL1β, interleukin 1 beta. Figure was created using BioRender.com.

The prevalence of MAFLD is rapidly growing; fibrosis is the major determinant for MAFLD progression. HSCs contribute to the majority of the ECM-producing profibrogenic MFBs during MAFLD. The cell–cell crosstalk between HSCs and other cellular components of the liver underlies HSC activation. Injured and stressed hepatocytes and activated macrophages play a crucial role in triggering and maintaining HSC activation, survival and proliferation by producing a wide range of profibrotic mediators (Figure 1). Hepatocytes may promote HSC activation both directly and indirectly and thereby NASH progression. Targeting pathways involved in metabolic stress of hepatocytes using GalNAc-coupled siRNA, which has received FDA approval for TTR amyloidosis [142], or by using pharmacological agonists/inhibitors might be a promising approach. Currently, obeticholic acid, a FXR agonist; PPAR agonist namely, Lanifibranor; glucagon-like peptide 1 receptor (GLP1R) agonist Semaglutide, thyroid hormone receptor β (THRβ) agonist Resmetirom and stearoyl-CoA desaturase 1 (SCD1) inhibitor Aramchol are in clinical trials for MAFLD treatment [143,144]. Targeting hepatocytes that initiate the cellular crosstalk to HSCs might provide a promising therapeutic strategy; however, further studies are required to establish the most relevant approaches in this regard.
